# Nylons with Applications in Energy Generators, 3D Printing and Biomedicine

**DOI:** 10.3390/molecules29112443

**Published:** 2024-05-22

**Authors:** Matteo Arioli, Jordi Puiggalí, Lourdes Franco

**Affiliations:** 1 Departament d’Enginyeria Química, Escola d’Enginyeria de Barcelona Est-EEBE, Universitat Politècnica de Catalunya, Av. Eduard Maristany 10–14, 08019 Barcelona, Spain; matteo.arioli@upc.edu (M.A.); jordi.puiggali@upc.edu (J.P.); 2 Barcelona Research Center in Multiscale Science and Engineering, Universitat Politècnica de Catalunya, Campus Diagonal-Besòs, Av. Eduard Maristany 10–14, 08019 Barcelona, Spain

**Keywords:** polyamide, nylon, triboelectric nanogenerators, 3D printing, catheters, sutures, biomedical, adhesive, membranes

## Abstract

Linear polyamides, known as nylons, are a class of synthetic polymers with a wide range of applications due to their outstanding properties, such as chemical and thermal resistance or mechanical strength. These polymers have been used in various fields: from common and domestic applications, such as socks and fishing nets, to industrial gears or water purification membranes. By their durability, flexibility and wear resistance, nylons are now being used in addictive manufacturing technology as a good material choice to produce sophisticated devices with precise and complex geometric shapes. Furthermore, the emergence of triboelectric nanogenerators and the development of biomaterials have highlighted the versatility and utility of these materials. Due to their ability to enhance triboelectric performance and the range of applications, nylons show a potential use as tribo-positive materials. Because of the easy control of their shape, they can be subsequently integrated into nanogenerators. The use of nylons has also extended into the field of biomaterials, where their biocompatibility, mechanical strength and versatility have paved the way for groundbreaking advances in medical devices as dental implants, catheters and non-absorbable surgical sutures. By means of 3D bioprinting, nylons have been used to develop scaffolds, joint implants and drug carriers with tailored properties for various biomedical applications. The present paper aims to collect evidence of these recently specific applications of nylons by reviewing the literature produced in recent decades, with a special focus on the newer technologies in the field of energy harvesting and biomedicine.

## 1. Introduction

Nylons, or aliphatic polyamides, are important industrial materials, valuable because of their good physical properties [1]. These polymers owe their characteristics to their ability to establish hydrogen bonds, which gives them properties of resistance to high temperatures and chemicals.

Nylons were first synthetized by the DuPont chemist Wallace H. Carothers [2] back in the 1930s mainly as a novel material used in fiber production [3].

Since then, the main applications of these polyamides have been in the textile industry [4] for the production of stockings, activewear, hosiery, lingerie, swimwear and outerwear. In textiles and domestic applications, nylon 6 and nylon 66 are the most exploited ones, with nylon 46 and nylon 56 [5] as potential alternatives. Other fields where nylons met application were in the production of parachutes, fishing nets, tents and ropes. In industrial applications, nylons find uses in the production of machine parts like bearings, wear pads, washers, bushings and sprockets [6]. Their remarkable properties make nylons meet most needs of many industrial sectors. Thus, nylons are increasingly employed as a substitute for metals and become an excellent solution that contributes to the durability of a product and to the reduction in overall operating costs of industrial machinery. In industries, the most used polyamides are nylon 66, nylon 610, nylon 11 and nylon 12 [7].

In recent years, the integration of nylons into cutting-edge technologies has expanded their role beyond traditional applications and ushered in an era of innovation in various fields. In particular, the emergence of triboelectric nanogenerators, advances in additive manufacturing and the development of biomaterials have highlighted the versatility and utility of these materials.

The use of nylons has been extended into the field of biomaterials, where their biocompatibility, mechanical strength and versatility have paved the way for groundbreaking advances in dental implants, catheters and sutures [8,9,10]. These materials exhibit favorable properties for mimicking the structural characteristics of natural tissues and organs, facilitating cell adhesion and proliferation and promoting tissue regeneration. Through innovative manufacturing techniques such as electrospinning and 3D bioprinting, researchers have used nylons to develop scaffolds, implants and drug carriers with tailored properties for various biomedical applications.

Known for their durability, flexibility and wear resistance, nylons have found wide acceptance in additive manufacturing processes. These technologies, often referred to as 3D printing, have revolutionized the manufacturing process by enabling the production of complex geometries with increased precision and efficiency. The compatibility of polyamides with selective laser sintering, fused deposition modelling, multi jet fusion and other 3D printing techniques has facilitated the creation of intricate components in industries ranging from automotive and aerospace to healthcare and consumer goods [11,12].

Triboelectric nanogenerators (TENGs) represent another frontier in the field of renewable energy and self-powered systems [13,14,15]. These devices harness mechanical energy from friction to generate electricity and offer a promising solution for powering small electronic devices and sensors. Nylons, with their excellent triboelectric properties and inherent flexibility, have emerged as key materials for the construction of efficient TENGs. Their ability to withstand repeated mechanical stress while maintaining electrical conductivity makes them ideal candidates for improving the performance and durability of these energy harvesting devices.

**Figure 1:**
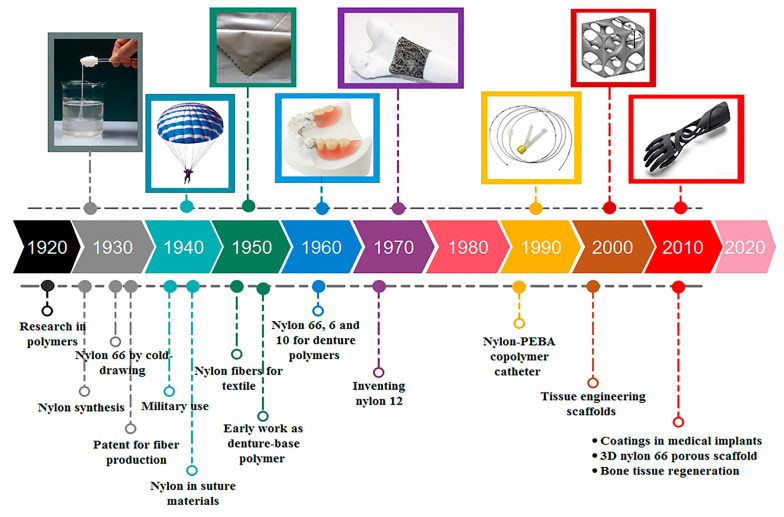
A timeline evolution of nylons, from the invention to the last application. Reproduced with permission from [16]. Copyright 2021 John Wiley & Sons Ltd.

A summary of the applications in different fields is shown in Figure 1, and Table 1 shows a list of commercial nylons, indicating the application and the start date of their use. Note that nylons started to be used in 3D printing and triboelectric nanogenerators in the 2000s and 2010s, respectively.

This review aims to provide an overview of some of these advanced technologies in which nylons are currently used and under continuous development and to explore the transformative impact of nylons within these dynamic technological fields. Special attention is given to the most recent applications of nylons in the field of energy harvesting and generation by exploiting the triboelectric effect and in their use as a material for the production of complex parts through addictive manufacturing 3D printing technologies, with a view also to their application in medicine as biomaterials.

## 2. Chemical Structure of Nylons

**Figure 2:**
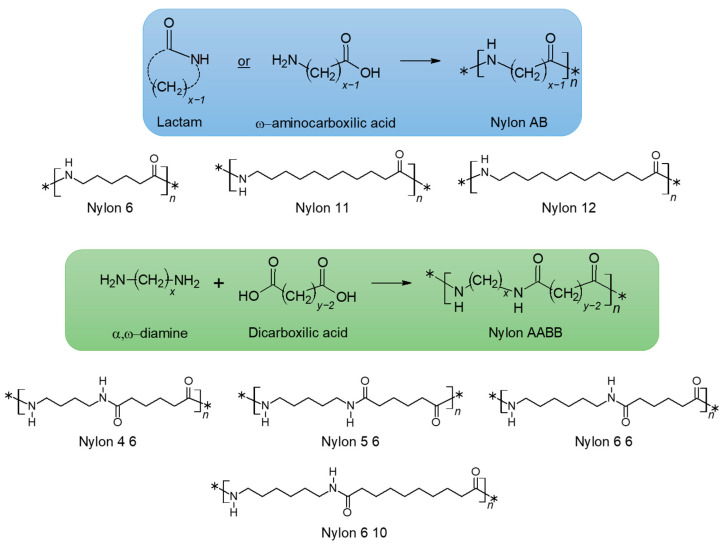
Nylons derived from unsubstituted nonbranched aliphatic monomers can also be named nylons *x* (**top lines**) for polyamides made from either an ω-aminocarboxylic acid or a lactam and nylons *x y* (**bottom lines**) for polyamides made from a diamine and a dicarboxylic acid. In the former classification, *x* indicates the number of carbon atoms in the repeating unit, while in the latter, *x* and *y* are the number of carbon atoms in the diamine and the dicarboxylic acid, respectively.

Nylons, or synthetic polyamides, can derive from the polymerization of ω-aminocarboxylic acids or lactams (nylons AB) or from the polycondensation of diamines with dicarboxylic acids (nylons AABB). A schematic representation of the different classification of nylons is depicted in Figure 2. In particular, general schemes of reactions for AB and AABB type nylons are represented, together with the repeating units of the nylon commercially available.

**Figure 3:**
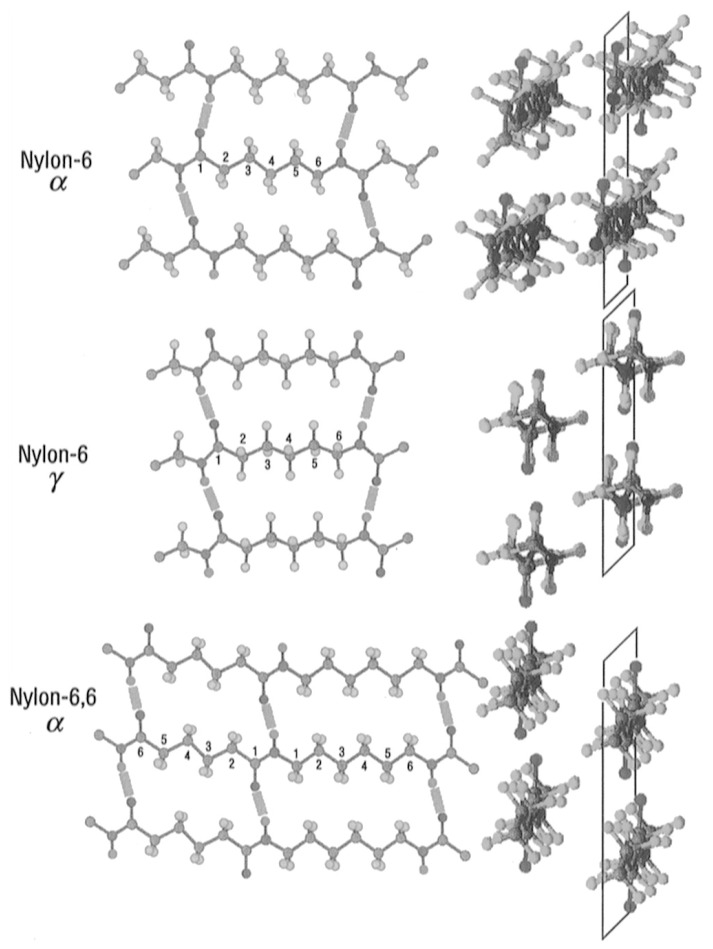
α (**top**) and γ (**center**) forms of nylon 6 and α form (**bottom**) of nylon 6-6 structures. Reprinted with permission from [38]. Copyright 1996 American Chemical Society.

The capability of amide groups to establish hydrogen bonds grants to these polyamides a crystalline structure characterized by polymorphism [34,35,36,37]. The most defined and concluded structures are the so-called α and γ forms, described by the different arrangement of the macromolecular chains in space and by the different development of the H-bonds (Figure 3). The α form is characterized by a triclinic unit cell (monoclinic in the variant β form) with hydrogen bonds formed along a single direction between the NH and CO groups of neighboring chains. The γ form, on the other hand, still present a single direction of development of the intermolecular hydrogen bonds, but it is characterized by a 60° rotation of amide groups from the typical sheet arrangement, which imparts a pseudohexagonal packing of the molecules.

A number of alternative structures have been postulated, according to the following different considerations: (i) Different length of the repeating units; (ii) Presence of odd-numbered carbon chains disturbing the geometries of the linear hydrogen bonds; and (iii) Establishment of hydrogen bonds in different directions. It has been proven [38,39] that there is a strong dependence on the final properties of the nylon depending on the adopted crystalline structure.

## 3. Nylon as Material for Triboelectric Application

### 3.1. Triboelectric Effect

The triboelectric effect refers to the phenomenon of contact-induced electrification, wherein a material becomes electrically charged upon contact with another material due to friction. This effect is a common occurrence in everyday electrostatics, with the charges acquired by a material determined by its relative polarity compared to the material it contacts [16,40].

**Figure 4:**
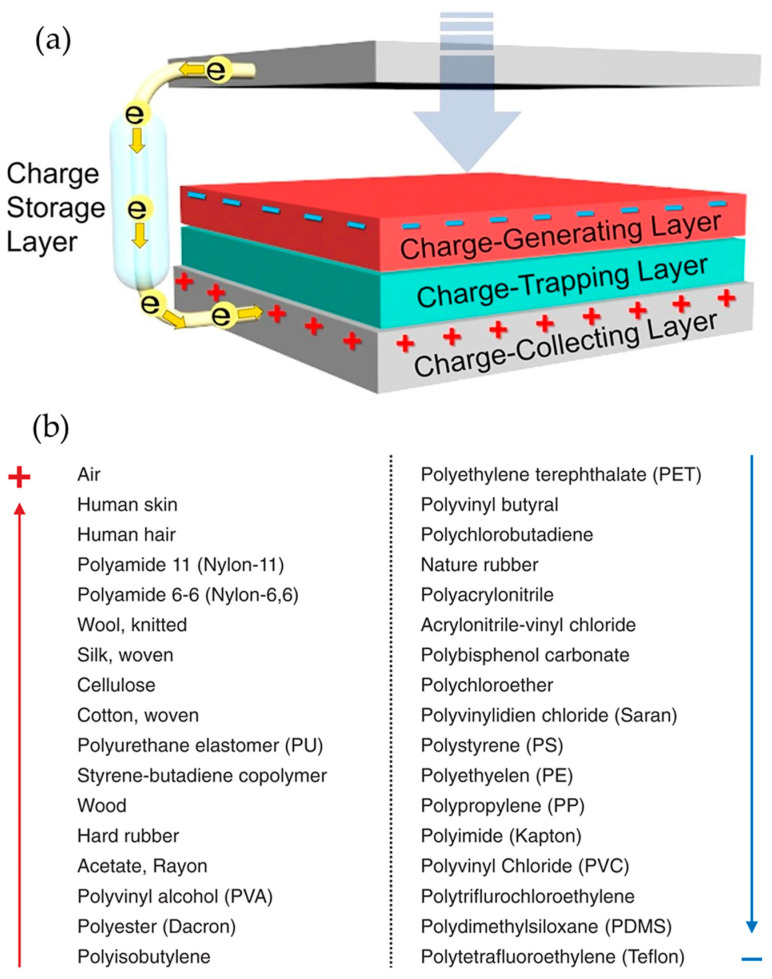
(**a**) Components of a triboelectric nanogenerator (TENG) affecting triboelectric power generation. The charge-generating layer (red), the charge-trapping layer (green), the charge-collecting layer (gray) and the charge-storage layer (white) can affect power generation in a TENG. Reproduced from [45]. (**b**) Triboelectric series. All known materials have different charge affinities and exhibit a contact electrification when two materials are in contact. Positive (or negative) means materials have a trend to lose (or gain) charge during contact motion. Reproduced from [43] Copyright 2020 The authors. *EcoMat* published by The Hong Kong Polytechnic University and John Wiley & Sons Australia, Ltd.

When two materials with different tendencies to give up or gain electrons come into contact and then separate, one material tends to lose electrons (becoming positively charged), while the other material tends to gain electrons (becoming negatively charged). A schematic representation can be seen in Figure 4a. This results in one material having a net positive charge and the other having a net negative charge. The degree of triboelectric charging depends on several factors, including the materials involved, their surface properties, the amount of surface contact and environmental conditions such as humidity. Materials with significantly different positions in the triboelectric series, which ranks materials based on their tendency to gain or lose electrons, will exhibit more pronounced triboelectric effects. Triboelectric charging has various practical applications, such as in static electricity generation, electrostatic precipitation (used in air purifiers and industrial applications) and even energy harvesting technologies, where mechanical motion is converted into electrical energy through triboelectric effects.

### 3.2. Triboelectric Nanogenerators

Inside this energy harvesting technology, triboelectric nanogenerators (TENGs) [41,42] are nanodevices able to effectively take advantage of the ambient mechanical energy by converting it into electricity. One of the key points in the production of these devices is the selection of the materials that constitute an active part of the assembly. The two materials must have the ability to either donate or gain electrons during contact, which provides them their properties as “tribo-positive” or “tribo-negative” materials, respectively [43,44]. A list of these two differentiated materials can be found in Figure 4b.

### 3.3. Nylons as a Component in Triboelectric Devices

Nylons exhibit specific crystal structures that are intrinsically linked to its ferroelectric behavior, a property crucial for enhancing the performance of triboelectric energy generators. Interestingly, the amide group in nylons possesses a permanent electric dipole moment, and the configuration of these dipole moments varies based on the number of carbon atoms [46,47]. Consequently, even-numbered nylons exhibit no net polarization due to the alternating arrangement of amide groups, while odd-numbered nylons demonstrate a net polarization owing to the alignment of dipole moments. This property categorizes odd-numbered nylons as “polar” and, consequently, as part of the group of “ferroelectric” polymers.

Focusing on nylon 11, it is notable to underline its extensive polymorphism that results in various crystal structures. The stable crystal structure, known as the α-phase, comprises stacked sheets of hydrogen-bonded chains. Other crystal phases of nylon 11, such as the β-phase, do not exhibit distinct properties and are of limited practical interest. The γ-phase, however, features slightly contracted molecular chains compared to the α-phase, with a pseudohexagonal crystal structure [48]. The δ-phase, resulting from the Brill transition, presents challenges in determining the arrangement of hydrogen bonding arrays, particularly above the transition temperature.

The ferroelectric behavior of nylon 11, particularly observed in the δ-phase, offers significant potential for applications in energy generation. High-voltage poling processes applied to cold-drawn or low-temperature-annealed samples (characterized by the so called δ_0_-phase) result in hysteresis loops in the displacement vs. electric field curve, indicative of ferroelectric behavior [49]. However, other phases of nylon 11, including α, its variant α_0_ and high-temperature-annealed δ_0_-phases, do not exhibit such behavior, highlighting the importance of specific crystal structures and hydrogen bonding arrangements [50].

Efforts to enhance the ferroelectric properties of nylon include mechanical drawing and solution processing methods [51]. Mechanical drawing, for instance, influences the ferroelectric behavior by inducing molecular orientation, thereby facilitating electric field-induced dipole reorientation. Similarly, solution processing, tailored with specific solvent systems, yields nylon films with high residual polarization values comparable to those obtained through complex melt-quenched-stretching processes.

**Figure 5:**
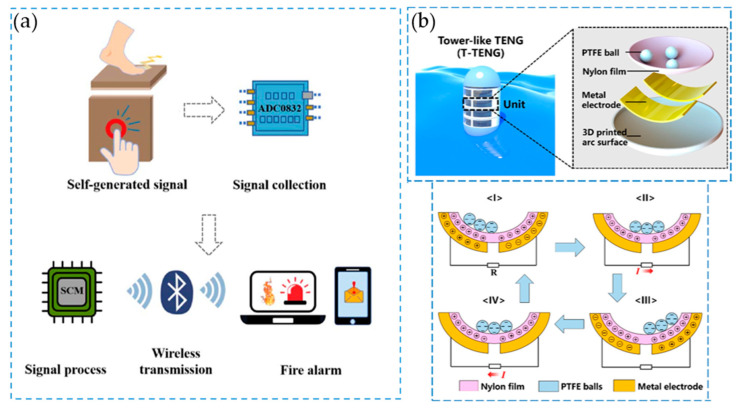
(**a**): Principles of fire alarm TENG-based system as described in [53]. Reproduced with permission from Elsevier. (**b**): Tower-like TENG, with a schematic view and the working principle. Modified from [64].

Nylons were recently used also as the main component in a fire-retardant TENG for fire alarms [52], where polyamide and polytetrafluoroethylene (PTFE) cotton fabrics were coupled, obtaining a flame-retardant, melt-dripping resistant material, with improved electrical performance [53] (for a schematic view, see Figure 5a). Recent studies are focused on the utilization of nylon surfaces as positive triboelectric materials. Singh et al. produced a new hybrid nanogenerator device by electrospinning Polyvinylidene fluoride (PVDF)/Molybdenum diselenide (MoSe_2_) fibers onto nylon fibers, obtaining an open circuit voltage peak of 113.6 V and a short-circuit current of 26.5 μA [54]. By the combination of amino-functionalized graphene oxide and nylon 12, as the positive material, with MoSe_2_/Polybutylene adipate terephthalate (PBAT) as the negative one, Gajula et al. produced a TENG with minimized charge loss [55]. Novel TENGs were recently made with silk-based materials, in which the poor mechanical properties and processability of the natural fiber have been overcome with a combination of PVA, Ag nanowires and nylon. The final TENG possessed wear-resistance, self-sensing capabilities and direct application in intelligent devices for monitoring human gestures [56]. By the combination of windproof outer textile and inner textile lining, Ag-coated nylon TENGs can be used as thermal insulation textiles [57]. In the field of electrochemical synthesis, TENG functionality has been recently investigated [58], in particular in the field of metal electrodeposition [59], electrochemical synthesis of nanoparticles and nanoclusters [60] and self-powered electrochemical processes [61,62]. In the application of polyamides in TENGs used for this purpose, Qiu Xiang Yang et al. developed a system based on the combination of an Epoxi/Polyamide (EP/PA) layer, coupled with PTFE ones. From the perspective of the triboelectric series, when PTFE comes into contact with EP or PA, PTFE readily gains electrons, while EP or PA swiftly loses electrons. EP and PA can undergo chemical cross-linking to form an EP/PA coating, which is widely utilized due to its exceptional anti-corrosive properties on metal substrates, resistance to abrasion and affordability. Thus, it is expected, and confirmed, that an EP/PA triboelectric layer would yield a sturdy and efficient TENG [63]. As a result, because of the excellent corrosion resistance and abrasion performance of the EP/PA coating, the W-TENG shows excellent durability in long-term work, where Ag NPs and O_2_ are successfully synthesized by this W-TENG-based electrochemical synthesis system.

Exploring the possibility of finding natural energy sources such as wave energy, different devices have been built to harvest this form of continuous energy. Xu et al. [64] and Xiong [65] developed different devices based on the possibility of harvesting wave energy from arbitrary directions. In particular, Xu et al. developed a system consisting of a tower-like TENG (see Figure 5b), which was constituted by different units of polytetrafluoroethylene balls and a three-dimensional printed arc surface coated with nylon. Again, by the combination of PTFE and nylon, Zhu et al. developed a different configuration plate TENG based on the pendulum effect (PP-TENG) [66]. The PP-TENG absorbs wave energy through the pendulum plate installed at the bottom of the device, which generates a swing effect. This drives the motion of the upper TENG power generation unit and generates a charge transfer on the surface of a film of polymer PTFE and nylon, enabling the possibility to harvest the irregular low-frequency energy typical of ocean waves. Another technique employed for the capture of these low-frequency sources is the employment of an escapement mechanism and frequency up-conversion device [67,68]. The escapement-mechanism-based triboelectric nanogenerator (EM-TENG), developed by Kwan-Woo et al. [69], consisted of a mechanical energy storage spring, an escapement mechanism and a torsional resonator to ensure stable operation and frequency up-conversion. In addition, the use of micro-patterned alternating dielectric surfaces of nylon and polytetrafluoroethylene (PTFE) along with a comb-type rotator greatly enhanced the output performance of the rotating EM-TENG, increasing the current density level by approximately 4.2 times compared to flat surfaces. In a work by Yan, a third configuration was developed [70]. The study introduced a novel concept of a swinging boat-type triboelectric nanogenerator designed for low-frequency wave energy harvesting. The swinging boat-type triboelectric nanogenerator (ST-TENG) consists of triboelectric electron generators equipped with electrodes and a nylon roller.

## 4. Nylon as Material for Additive Manufacturing Processes

**Figure 6:**
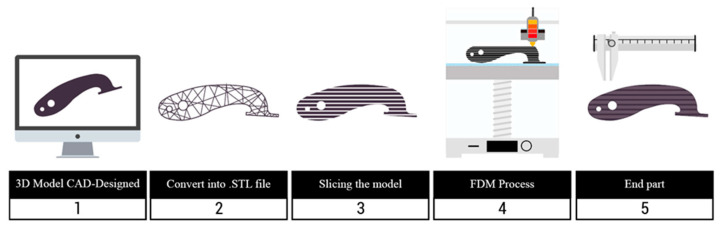
Schematic representation of the 3D printing process. Reproduced with permission from [71].

Additive manufacturing refers to a group of production processes, in which the final piece is obtained by the subsequent addition of a new material over a pre-formed one. These techniques require the use of 3D models designed by computer-assisted design software, which enables the production of pieces with medium to high complex geometry (Figure 6).

Fused deposition modelling (FDM) is a popular additive manufacturing (AM) technology, broadly referred to as 3D printing, in which the material is deposited layer by layer to create three-dimensional objects. Selective laser sintering (SLS) and multi jet fusion (MJF) are other two commonly used additive manufacturing techniques in the field of 3D printing [72,73,74]. Both SLS and MJF offer distinct advantages and are widely used in a variety of industries for purposes such as prototyping, customization and production [75]. Several studies have been performed to study how different printing parameters and conditions affect the final characteristics of the produced piece [76].

### 4.1. Fused Deposition Modelling

When it comes to FDM printing with nylon, there are several important aspects to consider [77]. The printability of nylon filaments typically requires a heated print bed to prevent warping during printing. In addition, an enclosed print chamber can help maintain stable printing conditions, as nylon tends to absorb moisture from the air, which can affect print quality. Additionally, the temperature settings must be changed since FDM printers using nylon work at higher temperatures than printers used to print PLA or ABS. Printing temperatures typically range from 220 °C to 260 °C for the nozzle and 60 °C to 100 °C for the heated bed, depending on the specific material and manufacturer recommendations. Printing at the correct temperature, ensuring proper cooling and optimizing print settings, such as layer height and extrusion multiplier, can help improve layer adhesion. Proper bed adhesion is critical when printing nylon to prevent warping and lifting of the printed part from the build platform. Techniques such as using a heated bed, applying adhesion promoters such as glue sticks or special bed adhesion sprays or using special build surfaces can help improve bed adhesion. Achieving strong layer adhesion is essential for producing durable parts with polyamides. Of particular interest is the usage of continuous-fiber-reinforced thermoplastic composites [78], in which thermoplastic materials have been reinforced with different types of fibers, including glass, carbon or Kevlar^®^. [79] Nylon is proving to be the optimum choice for gears, fan blades, sprockets, latches, manifolds and bearing surfaces due to its inherent self-lubricating properties, exceptional wear resistance and thermal stability [80].

Several works have been produced in recent years on the optimization of this technique with the use of nylon. Moradi et al. [81] conducted a statistical and experimental analysis on the different process parameters in the FDM of nylon 12. In particular, the infill percentage, thickness of layers, number of contours, maximum load at failure, elongation at break, part weights and load time were tested and optimized. The experimental results showed a remarkable trend: within the same printing parameters, adjusting the layer thickness from 0.2 to 0.3 mm led to the achievement of peak values for both the ultimate load (533 N) and elongation (595.5%). This change in layer thickness, while maintaining a constant print speed, resulted in an accelerated cooling rate, thereby increasing the strength while limiting the elongation. Furthermore, it became clear that film thickness has a definitive and consequential effect on overall print time, further emphasizing its importance in the additive manufacturing process.

**Figure 7:**
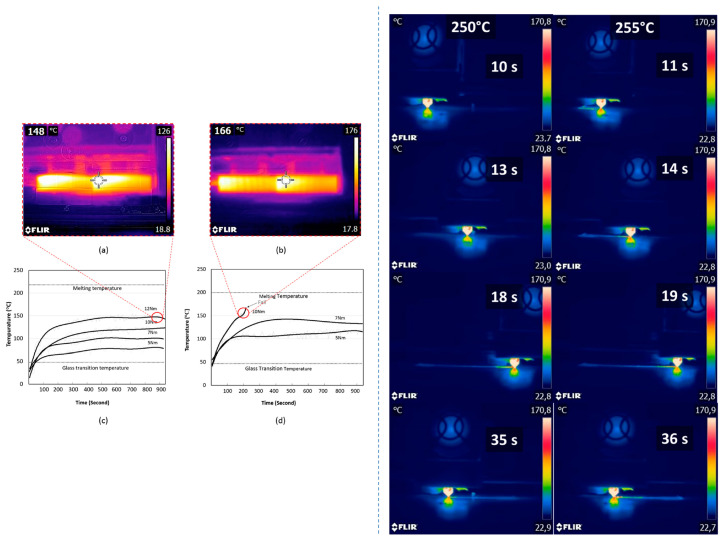
(**Left**): (**a**) Thermal image of nylon 618 gear with 12 Nm torque at 890 s. (**b**) Thermal image of nylon 6 gear with 10 Nm torque at 200 s. (**c**) Thermal behavior of nylon 618 gears at 1000 rpm. (**d**) Thermal behavior of nylon 6 gears at 1000 rpm. Reproduced with permission from [82]. Copyright 2019 Elsevier Ltd. (**Right**): Snapshots at different elapsed times of infrared recordings showing the evolution of nylon filament temperature during laying down process for two printing temperatures. It is possible to observe the more rapid cooling of the printed filament at 250 °C, compared to the one at 255 °C. Reproduced with permission from [83]. Copyright 2020 Wiley Periodicals LLC.

Comparisons were made between the literature data on injection-molded nylon 66, nylon 618 gears and five types of 3D-printed gears. In particular, nylon 618 showed superior performance under low to medium torque conditions compared to injection-molded gears. Interestingly, wear was only observed on the pitch line of the 3D-printed gears, whereas the nylon 618-printed gears showed localized melting of the tooth surface without material delamination. Conversely, the other four printed materials exhibited material delamination from the gear teeth. Differential Scanning Calorimetry (DSC) tests showed that nylon 66 and nylon 618 exhibited improved thermal properties, characterized by higher glass transition temperatures, melting temperatures and crystallinity compared to the other materials tested. Consequently, it was postulated that the superior friction and wear performance of nylon 618 compared to other printed materials was mainly due to its thermal behavior and the degree of sintering between each layer [82]. In Figure 7, top, it is possible to observe the infrared camera photograms and with the variation in temperature over time for the different processed materials.

By monitoring the microstructure, thermal behavior and tensile properties, Guessasma et al. [83] were able to determine the effect of the printing temperature on nylon, 3D-printed dog-bone-like specimens. Significant reductions in stiffness, strength and elongation at break were evident even at the highest printing temperature (255 °C, see Figure 7, bottom). The decrease in tensile performance was attributed to process-induced porosity, as revealed by X-ray micro-tomography imaging. This porosity was characterized by a large amount (39%), extensive connectivity (99%) and anisotropic distribution across the main spatial directions. The authors concluded that despite the low performances of the printed nylon, it remains a promising candidate due to its favorable fracture toughness results. Moreover, there is potential for enhancing both strength and stiffness by refining the control of process-induced porosity.

**Figure 8:**
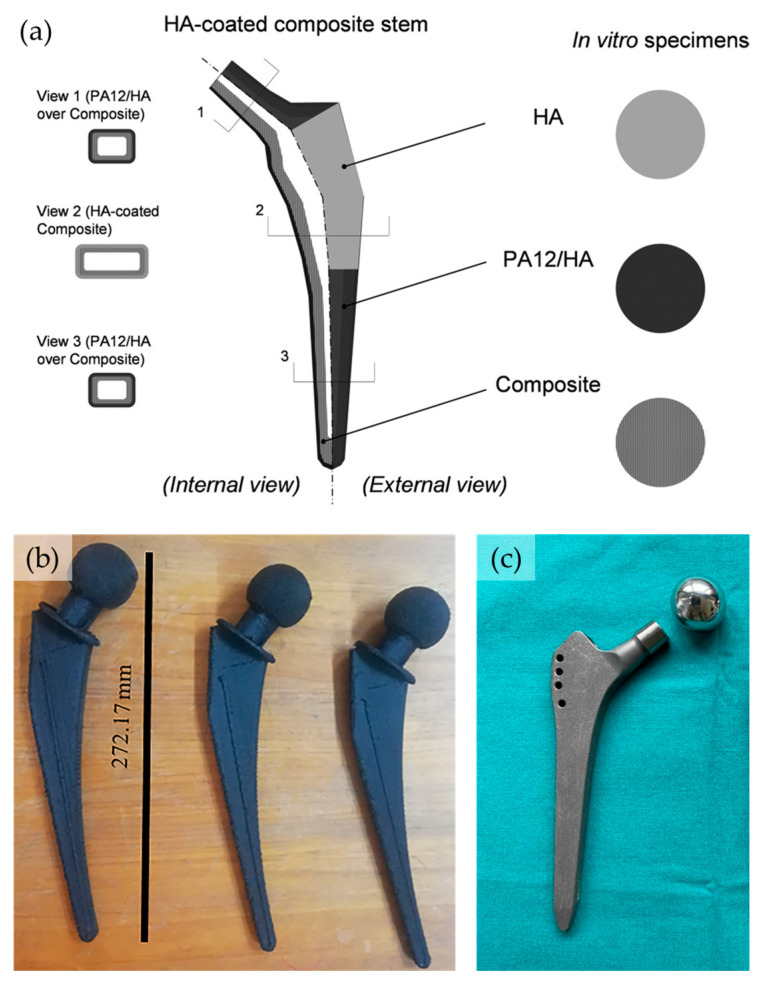
(**a**) Exploded view of the different part of a hip joint implant, showing the various materials composing the final prothesis (PA12: nylon 12; HA: hydroxyapatite). Reproduced with permission from [84]. Copyright© 2008 Wiley Periodicals, Inc. (**b**) A photograph of the FDM produced hip joint implant. Reproduced with permission from [86]. Copyright© 2022 Wiley-VCH GmbH. (**c**) A comparative picture of a titanium manufactured implant. Reproduced from [87].

In the production of 3D-printed hip joint implants, nylon 12 seems to have a clear and important role [84,85], particularly in a carbon-fiber-reinforced form (Figure 8). Again, with an optimized temperature of processing of 255 °C, Nyiranzeyimana et al. managed to produce FDM-printed hip joint implants, using a layer thickness of 0.3 mm and print speed of 50 mm/s [86]. The final pieces possessed a tensile strength, elastic modulus, percentage elongation and compressive strength of (71 ± 5) MPa, (7.6 ± 0.2) GPa, (1.9 ± 0.5)% and 135.8 MPa, respectively. As a great advantage of this applications, the final specimens possessed a density of 1.4 cm^3^ vs. 4.4 cm^3^ of a titanium-based prothesis.

### 4.2. Selective Laser Sintering

SLS uses a high-powered laser to selectively sinter powdered material, typically nylon or other polymers, layer by layer to form a solid 3D object [88]. Known for its ability to produce intricate geometries and functional components with exceptional accuracy, SLS is highly regarded in the industry [89,90].

**Figure 9:**
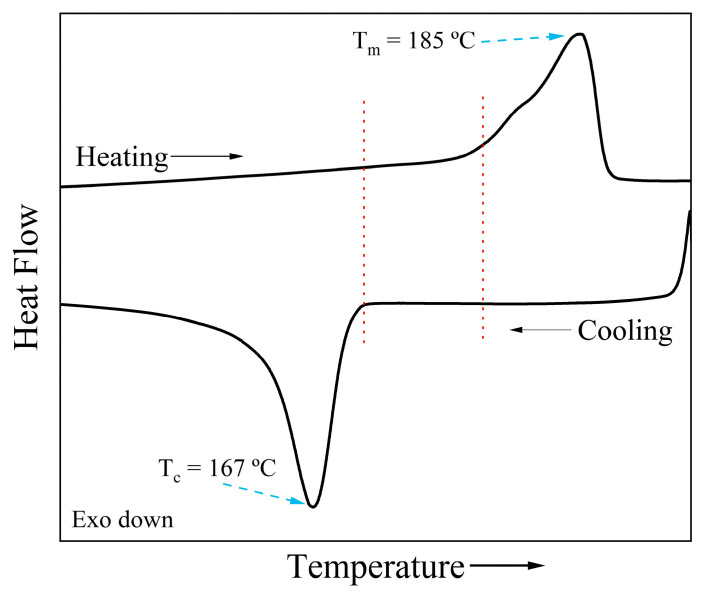
Heating and cooling runs of a nylon 11 sample, showing the wide thermal working range (between red dotted lines).

In this field, nylon 12 and nylon 11 are the most widely used polymers, because of the wide working temperature range between their onset melting and crystallization temperatures (see Figure 9) [91,92,93]. As a drawback, nylons, and polyamides in general, require higher working temperatures (from 180 °C to 280 °C) compared to other polymers used in this technique.

Ajoku et al. evaluated the build orientation of the parts having an effect on the mechanical properties of the nylon 12 pieces SLS produced. Specifically, the tensile tests showed a maximum difference of 16% and 11.2% in strength and modulus, respectively, for parts built in the *x*, *y* and *z* axes. Similarly, the flexure tests showed a maximum difference of 9.4% and 7% in strength and modulus, respectively, for the parts produced in the *x*, *y* and *z* axes. For the compression tests, there was a maximum difference of 3.4% and 13.4% in strength and modulus, respectively, for the parts produced in the *x*, *y* and *z* axes. The test parts built in the *x* axis orientation showed the highest strength and modulus values, while the parts built in the *z* axis orientation showed poor strength and modulus values [94].

Regarding the microstructure and chemistry changes, Esposito et al. [95] analyzed parameters such as melting temperature (T_m_), percentage of crystallinity (χ_c_), lamellae thickness (l_c_) and d_hkl_ spacing of nylon 11 as a function of the energy used by the laser in the SLS printing procedure. The T_m_ and the degree of crystallinity (χ_c_) decreased upon printing from 201 °C (χ_c_ = 38%) to 190 °C (χ_c_ = 21%). These results suggested that the printing conditions employed resulted in an irreversible alteration in the microstructure of the nylon 11 polymer.

Still in the area of the application of nylon 11 and 12 in SLS, a key point is the quality and characteristics of the employed powder. Studies focused on this were conducted by Pandelidi [96] and Verbelen [97]. In this way, powder availability, low initial zero-shear viscosity and minimal tendency to warp seems to raise the popularity of these polymers in the application in laser sintering. The post-condensation behavior appears to be pivotal in determining these characteristics. However, while beneficial for establishing such properties, this behavior adversely affects the powder’s recyclability and the consistency of part properties. Even the recyclability of the powder seems to play an important role.

Salazar et al. [98] performed a mechanical essay of petroleum-based nylon 12 and biobased nylon 11 processed by SLS, comparing various ambient conditions. It was demonstrated that nylon 11 exhibited higher toughness and ductility, especially at low temperatures, albeit with a mild decrease in modulus. Nylon 11 also showed the highest hydrothermal aging resistance. Moreover, the fatigue resistance of nylon 11 was superior under the studied conditions.

### 4.3. Multi Jet Fusion

**Figure 10:**
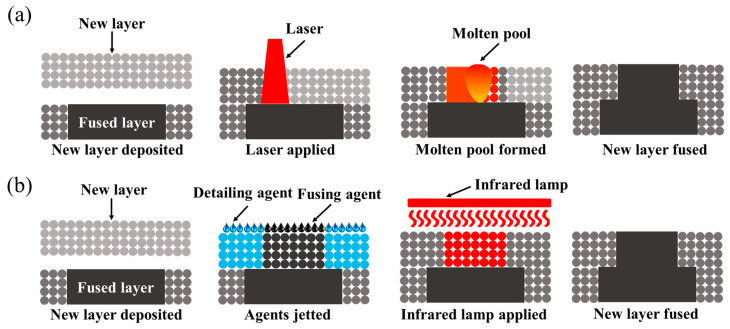
Comparative visualization of SLS (**a**) and MJF (**b**) processes. Reproduced with permission from [99].

Multi jet fusion (MJF) is a proprietary technology of Hewlett-Packard (HP) Inc; it uses an inkjet array to selectively apply fusing and detailing agents to a bed of powdered material, which is then fused by heat into a solid layer (Figure 10). This technology offers rapid production speed, cost-effectiveness and the ability to produce final parts and functional prototypes with high density, smooth surface finishes and dimensional accuracy. Regarding MJF-printed materials, several works have been published, aiming at the characterization and comprehensive understanding of the mechanisms and characteristics of the process and the produced parts.

Verbelen et al. [97] performed a thermal analysis on samples of virgin and recycled powders, noticing that while some thermal aging may have been observed in the nylon 11 powder used, as indicated by a slight increase in apparent melting temperature, this was not expected to affect processing, as it remained within the temperature limits of MJF processing. The small powder particles found in the nylon 11 powder mixture of 30/70 recycled/virgin were found to hinder flowability but did not inhibit the formation of a smooth surface to the same degree. In turn, the decreased flowability and increased surface fractal of the used nylon 11 powder potentially resulted in the formation of a rougher build layer surface. The authors concluded that this evidence showcases the successful processing of the recycled powder through MJF, affirming its feasibility and effectiveness within the manufacturing process.

Wei Shian published a study in which they explored the systematic characterization of materials used in MJF additive manufacturing, specifically focusing on polyamide 11 [100]. Through a comprehensive analysis, the researchers investigated the physicochemical properties of this material and evaluated the mechanical performance and print quality of the parts printed. The study revealed that nylon 11 powder exhibited irregular morphology and a wide particle size distribution, yet the printability remained largely unaffected. Tensile strength for nylon 11 was found to be highest in the Z-direction, while its flexural properties demonstrated similar anisotropy trends. Notably, nylon 11 displayed excellent wear resistance and smoother surface finishes.

**Figure 11:**
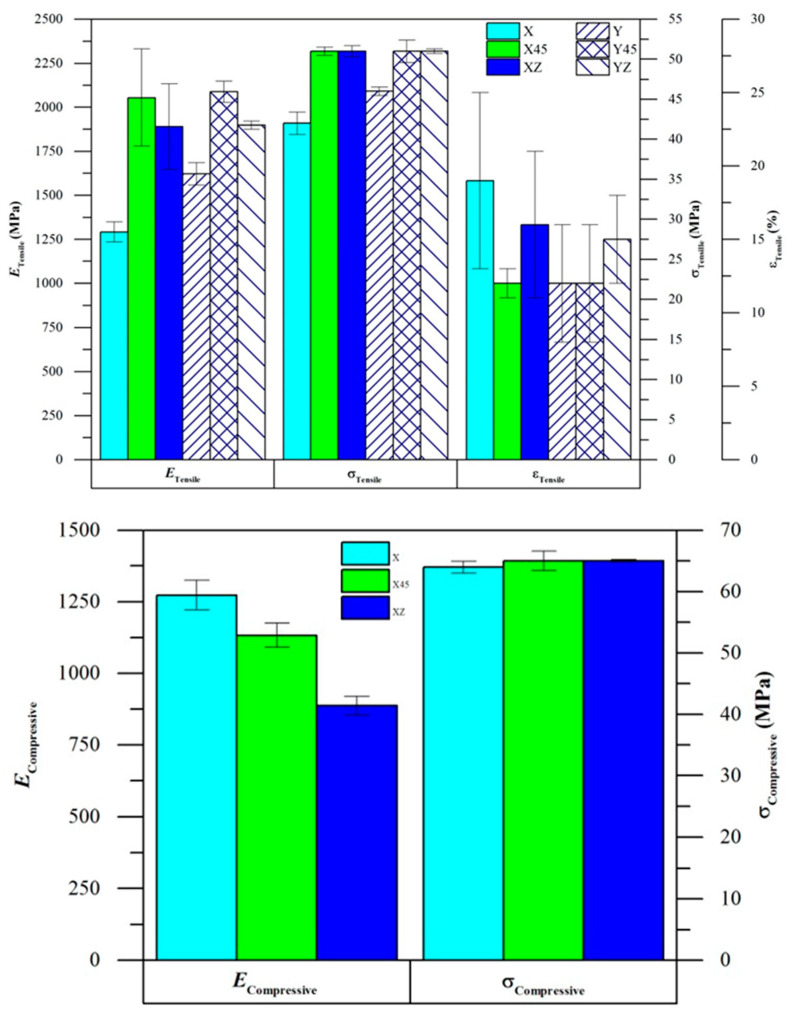
(**Top**): Tensile mechanical results over tests performed in different measuring directions. (**Bottom**): Compression mechanical tests results. Reproduced from [101].

In a more recent work [101], the researchers delved into the influence of print orientation on the tensile mechanical properties of MJF-manufactured nylon 12 parts. Through a series of experiments and analyses, they uncovered how the orientation of parts during printing affects their mechanical performance (see Figure 11). Vertical orientation is identified as favoring improved tensile properties due to more complete polymer powder fusion. Moreover, the study involved the calibration of an elastic–plastic with combined hardening (EPC) material model in Abaqus, which proved to be more accurate for finite element analysis (FEA) of additive manufactured nylon 12 compared to the commonly used EPI material model. This calibration process enhanced the understanding and prediction of mechanical behavior in MJF-printed parts, offering valuable insights for design optimization and performance enhancement in additive manufacturing applications.

A key point in the application of such a manufacturing technique is the approach that enables the possibility to predict and, later on, to control the crystallinity of the final piece. As mentioned in the paragraph devoted to the crystallinity of nylons, the final properties of the printed object depend on the crystalline structure of the nylon. A work by Le et al. [102] addresses this problem. In this work, a crystallinity prediction method based on machine learning for MJF-printed nylon 12 was presented, trained after a correct measurement of multiple specimens by DSC [103]. As a result, the authors explained that the formation of crystallinity was significantly affected by the duration of the first cooling stage, the temperature at the end of the printing process, the duration of extremely low cooling rate and the cooling condition of the second cooling stage. An interesting work was issued by Gavcar et al. [104], where the green tribological behavior of nylon 12 parts, manufactured by MJF with different build orientations, was evaluated by ball-on-disc tribological tests under different normal loads and lubricated environments.

The work of Sergio Morales-Planas [105] presented a comprehensive study of the sealing behavior for high-pressure applications, using MJF technology and nylon 12. The researchers examined the manufacturing process and the properties of MJF-printed parts, with a particular focus on their sealing properties. Through rigorous testing and analysis, they established manufacturing rules and guidelines for the development of pressurized products using MJF-printed nylon 12 components. The study demonstrated the superior performance of MJF technology in producing functional prototypes and final parts for fluid handling applications, outperforming traditional plastic molding methods. In addition, the effect of wall thickness and print orientation on tightness was thoroughly investigated, revealing significant correlations between these variables and the sealing integrity of printed parts. By validating tightness through the production of an ISO-compliant industrial ball valve, the potential of MJF technology for high-performance fluid handling components and its role in advancing additive manufacturing capabilities in various industries was highlighted.

In the study of the physical properties of the materials, Chen et al. [106] published a study delving into the viscoelastic–viscoplastic deformation behavior of MJF-printed nylon 12 through a combination of experimental [107] and numerical methods. Various multi-loading–unloading-recovery tests were performed to differentiate between viscoelastic and viscoplastic deformations. The experiments revealed that deformation was notably affected by the loading rate, loading history and control methodology. As deformation increased, porosity also increased due to extensive deformation of the local matrix and the merging of voids.

To address some of the issues of the MJF-printed polyamide 12 with respect to the lack in mechanical strength, Liu et al. [108] tested the use of glass fibers and annealing processes. In their work, a high-temperature (173 °C, near the onset melting temperature of nylon 12) annealing process was developed to remarkably enhance the mechanical strength. Specifically, the ultimate tensile strength (UTS)/tensile modulus of pure polyamide and reinforced specimens were increased by 20.8%/48.5% and 22.8%/30.6%, respectively.

Comparative studies regarding the use of nylon 12 in selective laser sintering and multi jet fusion sintering were conducted by Chao Cai [99], Xu [109] and Rosso [110]. By closely examining the morphologies, thermal properties and mechanical characteristics of nylon 12 parts produced through these two additive manufacturing techniques, valuable insights into their respective performances were gained. Both SLS and MJF employed nylon 12 powders with similar elliptical shapes, particle sizes, distributions and sintering windows. However, the surface roughness of MJF parts, particularly those treated with a detailing agent, exhibited significant improvement over SLS counterparts. This disparity in surface quality can be attributed to the differential effects of instant heating, with SLS demonstrating a higher degree of particle melting owing to the intense laser energy [12,111]. Despite the slightly lower mechanical properties observed in MJF parts compared to SLS, the remarkable difference in printing speed, with MJF being nearly ten times faster, underscores the distinct advantages offered by each technology. Further analysis of nylon 12 powder characteristics revealed that MJF powder boasted higher crystallinity and wider sintering windows, indicating its potential for optimized processing conditions. Interestingly, SLS-printed specimens demonstrated superior mechanical strength, particularly evident in parameters like Young’s modulus and elongation at break, possibly due to denser packing. Additionally, the comparative assessment of scaled-down pieces printed by both processes revealed that MJF exhibited higher printing accuracy, particularly in areas with intricate contours. These findings not only shed light on the nuanced performance differences between SLS and MJF [112] but also underscore the potential for further advancements in MJF technology through ongoing materials and process development initiatives. Another important study, conducted by Mehdipour et al. [113], investigated the anisotropic and rate-dependent mechanical properties of nylon 12 3D-printed pieces by comparison between the two techniques. Structural orientation was evaluated through studying the influence of different print orientations on the material properties, in both SLS and MJF printers. The results showed that print orientation has a notable effect on the mechanical properties of MJF specimens, while its influence is negligible for 3D printing with SLS.

## 5. Nylon as Material for Biomedical Devices

Polyamides are currently used for a wide range of biomedical applications [114] including bone regeneration scaffolds [115], tissue engineering [116] and membranes for protein separation [117]. In this section, the use of polyamide for the production of catheters, surgical sutures and dental implants is reviewed. Materials for medicine produced with nylons present all the advantages in terms of mechanical resistivity, bio compatibility and chemical inertness. On the other hand, nylons present a drawback due to the loss of some properties after moisture absorption [118,119]. For this reason, as explained later, studies are being conducted to address this issue.

### 5.1. Catheters

A catheter is a biomedical device, composed by a thin, flexible tube that is inserted into a body cavity, duct or vessel to allow drainage, injection of fluids or access to a specific area. Common materials for the production of catheters are silicone, polyurethane (PU), polyethylene (PE), polyvinylchloride (PVC), PTFE and nylon. Among them, nylon catheters are ideal for use as medical tubing because they are corrosion- and abrasion-resistant, lightweight and can withstand repeated stress over extended periods [120]. Currently on the market, the main available nylon-manufactured catheters are Rilsan^®^, by AesnoMed, Grilamid^®^ by Emsgrivory and Vestamid^®^, by Evonic. Different applications in catheters have been developed using nylons as the principal or reinforcing material [121,122]. Nylon 12 and 11 are widely and efficiently used in the treatment of Acute Ischemic Stroke [123,124] where the employment of catheter jackets takes advantage of the lower water absorption properties typical of these two linear polyamides. Newer applications rely on the design of composite materials, in which nylons are playing an important and key positioning role. Halim et al. [125] designed a nylon 11/montmorillonite nanomaterial with applications in angioplasty balloons. Another example comes from the production of novel renewable poly(ether-block-amide)s [121], used as a potential material for catheter balloon stents. Also, in the field of laparoscopic catheters, nylons present newer possibilities. A new active flexible endoscope holder [126] has been developed, characterized by a DNA-inspired helix-based structure with a wide angle and constant curvature bending and produced by selective laser sintering of nylon 11. A new balloon catheter, uniquely reinforced with nylon micromesh, was studied and developed by Kanji [127]. This type of catheter facilitates mitral commissurotomy, a cardiac procedure, without the need for thoracotomy, a major surgical procedure. In particular, it was used successfully to treat five out of six patients diagnosed with mitral stenosis.

### 5.2. Sutures

Nylons are extensively being used as a primary material for sutures [128,129]. In this field, linear polyamides represent examples of non-absorbable sutures, (see Table 2) in which other materials, like polyolefins or stainless steel thread, are present too. 

**Figure 12:**
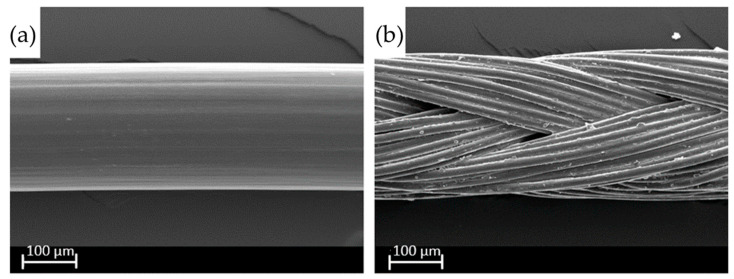
SEM micrographs of nylon monofilament (**a**) and a polyester multifilament (**b**) surgical sutures. Reproduced from [135].

Moreover, sutures based on polyamides have unique characteristics due to their mechanical resistance, which allow the production of multifilament and braided sutures, widening the range of applications [134]. Monofilament sutures consist of a single filament. This allows the suture to pass through tissue more easily. Multifilament braided sutures consist of several small threads braided together. This can provide greater security but at the cost of increased potential for infection [135]. Scanning electronic micrographs can be seen in Figure 12.

Among the others, nylon 6 and nylon 66 are the most exploited ones [136,137,138]. These sutures are available in a variety of sizes and configurations, allowing surgeons to select the most appropriate option for specific tissues and procedures.

Nylon sutures are preferred for their strength, which provides effective wound closure and promotes optimal healing [139]. In addition, their smooth surface reduces tissue trauma during insertion and removal, minimizing the risk of infection and inflammation [134,140,141,142,143]. In addition, nylon sutures are highly resistant to degradation in the body, ensuring long-term wound support until healing is complete. Overall, nylon sutures are a valuable tool in modern surgical practice, contributing to successful outcomes and patient well-being [144,145]. In between the different surgical fields, nylon sutures are used in abdominal fields [146,147], ophthalmology [148,149,150] and tendons/nerves repair [151,152,153,154]. Recent studies are focused on the enhancement of the antibacterial potency of nylon-based sutures. Syukri et al. performed a functionalization of nylon sutures through an in situ deposition of biogenic Au nanoparticles. The treated sutures demonstrated bactericidal effects on Gram-positive (*S. aureus*) and Gram-negative (*E. coli*, *P. aeruginosa*, *A. baumannii* and *K. pneumoniae*) wound pathogens with more than 99.9% reduction [155]. The deposition in situ of the silver nanoparticle did not affect the mechanical properties of the sutures and enhanced the antibacterial and wound healing properties. In another published work by Meghil, a novel quaternary ammonium compound, K21, was used as a functional coating over different suture materials, including nylons [156]. Results showed that the growth of *P. gingivalis* and *E. faecalis* was inhibited by K21 at concentrations ranging from 5% to 25% when applied to different suture materials. Mohammadi et al. [157] studied another coating for nylon sutures based on hyaluronic acid and chitosan with a layer-by-layer assembly. These polycationic and polyanionic materials were proven to form a polyelectrolyte matrix that can enhance the antibacterial property of the treated sutures by a controlled release of the coating materials.

### 5.3. Dental Implants

Nylons are used in dental implants, particularly in denture components, as an alternative to poly(methyl methacrylate) (PMMA) and acrylic resins, ref. [158], a material that can produce allergic responses. Dentures are primarily used to anchor artificial teeth and gums to the denture [159]. Despite this fact, acrylic resins are still used in the field, particularly when reinforced with nylon [160,161]. Dental nylon has unique qualities by the combination of flexibility and softness with strength and resistance to damage. This material is well tolerated by allergy sufferers. On the other hand, nylon dentures are able to completely imitate the colors of the oral cavity, without losing their original appearance. Additionally, nylon is not prone to retaining odors, because it does not collect microbes. Specifically, nylons are used as supports in mesh-forms [162], in an electrospun nanofiber form [163] or as fiber produced via the wet spinning method [164]. These systems are proven to be effective and durable [165].

An interesting application in the field of the electro-mechanical properties of polyamides has been explored by Baojin Ma [166]. They produced highly cytocompatible, piezoelectric nylon-11 nanoparticles, capable of providing physical signals to different stem cell during their differentiation processes. As a result, the osteogenic differentiation of dental pulp stem cells was found to be actively promoted by these nanoparticles, after mediation with ultrasounds, providing regulation by noninvasive stimulation.

### 5.4. Scaffolds for Tissue Engineering

The combination of a polymeric matrix with a bioactive material has been used as an effective composite for application in the tissue regeneration/engineering field. Examples are the combination of bioactive ceramic hydroxyapatite with polyethylene glycol [167] or polylactic acid [168].

Following this route, nylon-based composite scaffolds have been explored for various purposes, including skin regeneration, bone repair, cartilage restoration and vascular tissue engineering [29]. For instance, nylon-based scaffolds seeded with appropriate cell types have shown promise in promoting tissue regeneration in animal models, offering a potential solution for addressing injuries or diseases that compromise tissue function [169,170]. In a work by Wang et al. [171], mesenchymal stem cells (MSCs) derived from the bone marrow of neonatal rabbits were cultured, expanded and seeded on nano-hydroxyapatite/nylon 6 scaffolds. The results confirmed that scaffolds based on the composite ceramic/polyamide material are biocompatible and have no negative effects on the MSCs in vitro.

Other types of scaffold have been prepared by flock technology [172], in which short fibers of flocks (nylon 6 6 precision cut flock) were applied vertically on a substrate and coated with a flocking adhesive [173]. Over this matrix, embedded with collagen type I, articular chondrocytes were seeded, to promote the growing of articular cartilage.

Another generation of material composite scaffolds based on nylon are those integrating polyamides with chitosan [174,175]. This biopolymer, produced from the deacetylation of naturally occurrent chitin, has been found to possess non-toxic, antibacterial, anti-oxidant and anti-fungal properties. The combination of these two materials allows the production of mechanically resistant and bioactive scaffolds, for use in tissue engineering. Shrestha et al. [176] reported that this type of scaffold could increase osteoblastic MC3T3-E1 cell growth, adhesion, differentiation and proliferation, to enhance osteogenic capabilities. Chia-Hsiang Yen et al. [177] developed a platform for a continuous cell production, in which, by means of a change in the medium pH, adipose-derived stem cells were mass-produced with sustained regenerative capacity.

Despite promising advancements, challenges remain in optimizing the properties of nylon-based materials for specific tissue engineering applications. Issues such as degradation kinetics, immune response and long-term biocompatibility need to be carefully addressed to ensure the safety and efficacy of these constructs in clinical settings [178,179].

## 6. Nylon as Material for Other Applications

### 6.1. Hot Melt Adhesives

Adhesives that solidify from a molten state by cooling are commonly referred to as hot melts. They belong to the category of thermoplastic adhesives, which soften and liquefy when heated and solidify when cooled [180]. For effective bonding, the molten hot melt must maintain a low enough viscosity to ensure proper wetting, while avoiding rapid cooling that could hinder surface coverage on the substrate. Immediate bonding of substrates is required once the hot melt is applied in its molten form.

**Figure 13:**
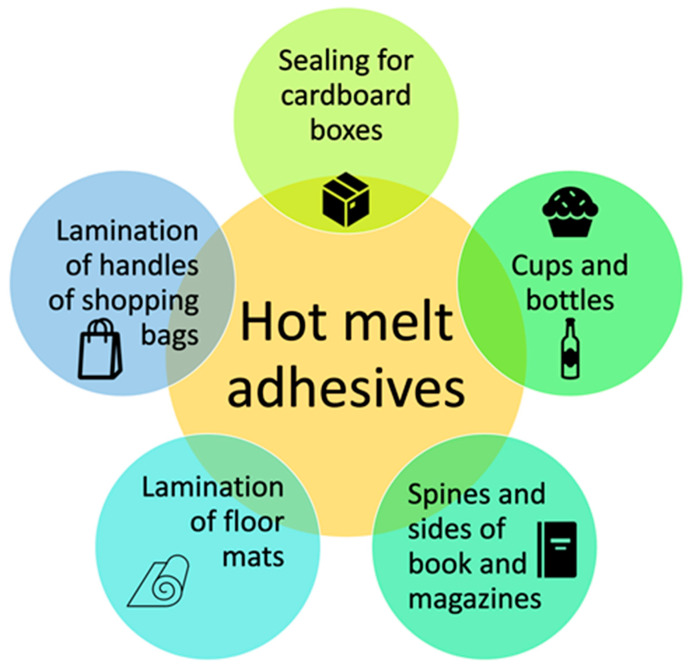
Common applications of hot melt adhesive.

After curing, the tackiness of the hot melt adhesive can vary depending on its specific formulation. The multiple fields in which these materials are employed are varied, mainly for the production of commonly used objects, such as books, cardboards and boxes (see Figure 13).

A wide number of different patents, describing the use of polyamides in application as hot melt adhesives, were issued in recent decades [181,182,183,184], providing a range of industrial solutions for extensive application across various industries. In packaging, they are commonly utilized for sealing cartons, boxes and envelopes. They play a crucial role in product assembly processes, bonding components in industries ranging from automotive and electronics to furniture manufacturing. Moreover, hot melts are employed in woodworking tasks, facilitating the bonding of veneers, laminates and edge banding and assembling wooden components. Recent works have been published on the enhancement of the properties of nylons in their application as hot melt adhesives. Jin et al. [185] successfully prepared an adhesive based on a copolyamide from aminoundecanoic acid, caprolactam and nylon 66 salt. Again, using copolymers of nylons, different materials have been produced. Zhou et al., by the combination of nylon 6, 66 and 1010, developed a melt adhesive for glutinous lining and PET/cotton fabrics [186], while in a work by Xue [187], using a modified version of this copolyamide with 6, 66 and 510, a successful adhesive for aluminum and low-surface-energy coating was reported. By the aggregation of multiwalled carbon nanotubes in a mixture of co-polyamides by Latko-Durałek et al. [188], the rheological behavior and electrical properties of the final hot melt adhesive was characterized and tuned. In 2020, nylon 6 containing LiBr was shown to have strong lap shear strength with a metal plate immediately after hot-melt adhesion [189], properties which can be employed for various applications such as high-performance masking films.

### 6.2. Membranes for Water Purification

Membranes for water separation are a critical component in various processes aimed at purifying water or separating specific components from water streams [190]. These systems are engineered to allow the passage of certain molecules or particles while blocking others, based on factors such as size, charge or chemical properties [191]. Nylon membranes are commonly used in water purification systems due to their excellent properties, such as chemical resistance, durability and the ability to produce precise pore sizes. These materials are often employed in various filtration processes, including microfiltration, ultrafiltration and nanofiltration, depending on the pore size and filtration requirements [192].

In water purification, nylon membranes can effectively remove particles, bacteria, viruses and other contaminants from water, making it safe for consumption or further treatment. They are widely used in both residential and industrial settings, including in point-of-use water filters, desalination plants and wastewater treatment facilities. The pore size of the nylon membrane can be tailored to specific applications, allowing for the selective removal of contaminants while retaining essential minerals and nutrients in the water [193]. Additionally, nylon membranes can be easily manufactured in different forms such as flat sheets, hollow fibers or spiral-wound modules, providing versatility in system design and implementation.

On the technology of polyamides as water purification membranes, different works have been published. The majority of them aims to find solutions for the maximization of purified water production [194]. This involves a trade-off between the permeability and the selectivity of a membrane, being a compromise between a huge volume of obtained water and its high quality. The more recent works deal with the functionalization of polyamide membranes by the application of thin-film composites. Being characterized by high permeability and selectivity, these systems can address the benchmarks of the problem [195,196,197]. These composite membranes consist of a non-woven fabric support, a porous middle polymer layer and a thin (<500 nm) and highly cross-linked polyamide layer to provide selectivity [198]. Among these materials that, by a chemical or physical application to the polymeric membrane, constitute the thin-film composite, it is possible to find metal–organic frameworks (MOFs) [199,200,201]. MOFs refer to a type of hybrid organic–inorganic solid material consisting of metal ions or clusters coordinated by organic moieties [202]. It has been reported that the implementation of this organic–metallic agent can positively affect the permeability of the membrane [203], enhancing the amount of produced water without the cost of reduction in selectivity [204,205].

Another important functionalization has been provided by the integration of graphene oxide in the membrane as a thin-film compound [206]. In the membrane separation area, graphene with controlled pores is believed to form the ultimate thin membrane for fluid or gas separation [207]. Its application in water purification membranes has been studied [208,209,210]. The insertion of graphene-oxide in nylon membranes, in the form of nanosheets, has a dual effect: it increases the selectivity of the membrane, obtaining a pure stream of purified water without reducing the flow; also, it acts as a chlorine barrier for the underlying polyamide membrane, resulting in a profound suppression of the membrane degradation in salt rejection upon chlorine exposure [211].

## 7. Conclusions

This review gathers studies on the technological and modern applications in which nylons are the main or constitutive material. Since their invention nearly 100 years ago, these polymers have proven to be important technical materials with a wide range of applications. Still widely used in the fields for which they were created as synthetic fibers, these polyamides demonstrate a wide range of applications, from the production of biomaterials to constituting the main material in advanced devices such as triboelectric nanogenerators or dental and femoral prostheses printed by additive manufacturing processes. The employment in commercial and industrial uses like hot melt adhesives and membranes for water purifications confirms the good employability of these polymers in different spheres.

Taking into account the analyzed bibliography, some considerations can be formulated. The largest number of linear polyamides produced and used are those belonging to the group of traditional nylons, including nylon 6, nylon 6 6, nylon 12 and nylon 6 10. There is an increasing interest in the use of nylon 11, especially for 3D printing technologies. Other nylons, such as nylon 5 6, nylon 5 10 or nylon 4 6, are industrially produced but are still limited to specific applications, replacing the best known and most widely used.

The application of nylon in triboelectric nanogenerators is quite new, as is the implementation in additive manufacturing processes. For these two topics, the optimization of the conditions, characterization and final design are still under complete study. In the case of triboelectric devices, different configurations are being studied with the main objective of maximizing the energy gain. As for 3D-printed parts, their optimization is based on the fact that they must simulate the mechanical properties of other non-plastic materials, such as ceramics or metals. The main advantages are the reduction in weight and the possibility to create complex structures.

The literature data indicate that a lot of research is being produced to improve the properties of nylon by increasing its applicability, particularly in the fields of surgical sutures and membrane separation technology: in the former case, by improving the antibacterial character, while in the latter, by increasing the permeation and selectivity.

Great efforts are being made to produce biobased monomers for the synthesis of nylon from bio resources [212]. Some of these monomers are obtained from vegetable oils like castor oil for adipic acid [213] and sebacic acid [214] 1,5-pentanediamine [215]. Thus, several all-biobased nylons, such as nylon 11, nylon 10 10 and nylon 4 10, or partly biobased nylons, such as nylon 6 10 and nylon 10 12, with excellent engineering applicability, have been already commercialized [216,217]. Nevertheless, although the literature produced on these aspects is very fruitful, these polymers are still on their way to being widely industrially produced.

## Figures and Tables

**Table 1:** Summary of commercial nylons with their most common applications.

Application		Polyamide	Decade of the First Application or Publication
Textile fibers		Nylon 6, nylon 5 6, nylon 6 6, nylon 10	1940s [17,18]
Industrial fibers		Nylon 6, nylon 5 6, nylon 6 6, nylon 5 6, nylon 6 10	1940s [19]
Automotive (injection-molded pieces)		Nylon 11, nylon 12, nylon 4 6	1960s [20]
Water purification membranes		Nylon 6	1960s [21,22]
Hot melt adhesive		Nylon 6, nylon 6 6	1970s [23]
Biomaterial	Sutures	Nylon 6, nylon 6 6	1940s [24,25]
	Catheters	Nylon 6, nylon 6 6	1960s [26,27]
	Dental implants	Nylon 6, nylon 6 10	1940s [28]
	Tissue engineering scaffolds	Nylon 6, nylon 6 6	2000s [29]
3D printing	SLS	Nylon 11, nylon 12	2000s [30]
	FDM	Nylon 11, nylon 12	2010s [31]
	MJF	Nylon 12	2010s [32]
Triboelectric nanogenerators		Nylon 11, nylon 6 6	2010s [33]

**Table 2:** Commercial non-absorbable surgical sutures, along with their constituent material and the main characteristics [130,131,132,133].

Tradename	Material	Description	Manufacturer
Ethilon™	Nylon 6	Monofilament	Ethicon, Raritan, NJ, USA
Kruuse Nylon	Nylon 6 + Nylon 6 6	Monofilament	Krause, Langeskov, Denmark
Seralon	Nylon 6	Monofilament	Serag Wiessner, Naila, Germany
Aragó Nylon	Nylon 6	Monofilament	Laboratorio Aragó, Barcelona, Spain
Resolon	Nylon 6 + nylon 6 6	Monofilament	Resorba, Nuremberg, Germany
Monosof™/ Dermalon™	Nylon 6 + Nylon 6 6	Monofilament	Covidien, Tokyo, Japan
Dafilon^®^	Nylon 6 + Nylon 6 6	Monofilament	B. Braun, Melsungen, Germany
Nurolon™	Nylon 6	Multifilament (braided)	Ethicon, Raritan, NJ, USA
Supramid^®^	Nylon 6 + Nylon 6 6	Multifilament (core-sheath) Monofilament	B. Braun, Melsungen, Germany
Trelon^®^	Nylon 6 + Nylon 6 6	Multifilament (braided) Coated	B. Braun, Melsungen, Germany
